# Nicotinamide phosphoribosyltransferase leukocyte overexpression in Graves’ opthalmopathy

**DOI:** 10.1007/s12020-015-0855-8

**Published:** 2016-01-14

**Authors:** Nadia Sawicka-Gutaj, Bartłomiej Budny, Ariadna Zybek-Kocik, Jerzy Sowiński, Katarzyna Ziemnicka, Joanna Waligórska-Stachura, Marek Ruchała

**Affiliations:** Department of Endocrinology, Metabolism and Internal Medicine, Poznan University of Medical Sciences, Poznań, Poland

**Keywords:** Graves’ disease, Graves’ ophthalmopathy, Graves’ orbitopathy, Visfatin, Nicotinamide phosphoribosyltransferase, Pre-B cell colony-enhancing factor

## Abstract

To investigate the role of NAMPT/visfatin in euthyroid patients with Graves’ disease without (GD) and with Graves’ ophthalmopathy (GO), we analyzed *NAMPT* leukocyte expression and its serum concentration. This was a single-center, cross-sectional study with consecutive enrollment. In total, 149 patients diagnosed with Graves’ disease were enrolled in the study. We excluded subjects with hyper- or hypothyroidism, diabetes mellitus, other autoimmune disorders, active neoplastic disease, and infection. The control group was recruited among healthy volunteers adjusted for age, sex, and BMI with normal thyroid function and negative thyroid antibodies. Serum levels of visfatin, TSH, FT4, FT3, antibodies against TSH receptor (TRAb), antithyroperoxidase antibodies, antithyroglobulin antibodies, fasting glucose, and insulin were measured. *NAMPT* mRNA leukocyte expression was assessed using RT-qPCR. NAMPT/visfatin serum concentration was higher in GD (*n* = 44) and GO (*n* = 49) patients than in the control group (*n* = 40) (*p* = 0.0275). *NAMPT* leukocyte expression was higher in patients with GO (*n* = 30) than in GD patients (*n* = 27) and the control group (*n* = 29) (*p* < 0.0001). Simple linear regression analysis revealed that NAMPT/visfatin serum concentration was significantly associated with GD (*β* = 1.5723; *p* = 0.021). When *NAMPT* leukocyte expression was used as a dependent variable, simple regression analysis found association with TRAb, fasting insulin level, HOMA-IR, GD, and GO. In the stepwise multiple regression analysis, we confirmed the association between higher serum NAMPT/visfatin level and GD (coefficient = 1.5723; *p* = 0.0212), and between *NAMPT* leukocyte expression and GO (coefficient = 2.4619; *p* = 0.0001) and TRAb (coefficient = 0.08742; *p* = 0.006). Increased * NAMPT* leukocyte expression in patients with GO might suggest a presently undefined role in the pathogenesis of GO.

## Introduction

Visfatin, primarily identified as a cytokine pre-B cell colony-enhancing factor (PBEF), also known as nicotinamide phosphoribosyltransferase (NAMPT), has nomenclature that reflects its complex functional aspects [1]. NAMPT is an intracellular enzyme essential for ATP synthesis. NAMPT/visfatin is secreted by adipose tissue and leukocytes as an extracellular protein, but its main source is still elusive [2, 3]. The protein is involved in metabolic regulation and its elevated levels were observed in patients with diabetes mellitus type 2 and obesity [4, 5]. Moreover, both hypo- and hyperthyroidism influence NAMPT/visfatin level [6, 7]. As a cytokine, it promotes inflammation and was found to be elevated in sepsis, autoimmune disorders and in low-grade inflammation, i.e. atherosclerosis [8]. Finally, NAMPT has an anti-apoptotic activity in many cancers, and we have recently found its overexpression in thyroid malignancies [9–11]. What more, we demonstrated that up-regulation of NAMPT correlated with tumor stage and lymph node invasion.

NAMPT/visfatin, a potent mediator of inflammation, was increased in several autoimmune diseases, such as, rheumatoid arthritis, systemic lupus erythematosus, ulcerative colitis, Crohn’s disease, and psoriasis [12–17]. Furthermore, some authors found that NAMPT/visfatin level positively correlated with disease severity in psoriasis and rheumatoid arthritis [13, 15, 16, 18].

A few studies evaluated the relationship of thyroid function and NAMPT/visfatin, but the results are inconclusive, possibly due to the heterogeneous etiology of thyroid disorders (autoimmune and non-autoimmune) [19].

Graves’ disease is an autoimmune disease. About 25–50 % of these patients develop Graves’ orbitopathy that might lead to irreversible ocular changes resulting in disturbed vision, changed facial appearance, and a significantly decreased quality of life [20]. The pathogenesis of Graves’ orbitopathy is unresolved so far, and many distinct factors are postulated to be involved (endogenous, genetic, environmental) [21–23]. Recently published study suggested the association between Graves’ orbitopathy prevalence and severity and diabetes mellitus type 2 with accompanying overweight [24].

NAMPT/visfatin is an adipocytokine that may link metabolism with autoimmunity. Therefore, we aimed to investigate the role of NAMPT/visfatin in euthyroid patients without (GD) and with orbitopathy (GO). In this study, we analyzed *NAMPT* leukocyte expression and its serum concentration.

## Materials and methods

### Study design and patients’ enrollment

This was a single-center, cross-sectional study with consecutive enrollment. In total, 149 patients with diagnosis of Graves’ disease were referred to the Department of Endocrinology, Metabolism and Internal Medicine or its outpatient clinic. The study was conducted between September 2013 and August 2014. Exclusion criteria were hyper- or hypothyroidism, diabetes mellitus, other autoimmune disorders, active neoplastic disease, and infection.

Anthropometric, clinical, and laboratory data were collected. An ophthalmological evaluation of GO patients was carried out according to the EUGOGO guidelines [25]. GO severity was assessed and clinical activity score (CAS) was used to measure the GO activity (CAS ≥ 3/7 indicates active GO). All patients with GO had MRI scan of orbits that confirmed the diagnosis and the activity of the thyroid associated ophthalmopathy. Forty healthy volunteers (29 women and 11 men) were recruited to serve as controls. Median age of the control group was 43.5 years (IQR 37.5–55 year) and median BMI was 23.3 kg/m^2^ (IQR 20.85–26.2 kg/m^2^).

The Ethics Committee at Poznan University of Medical Sciences approved the study and an informed written consent was obtained from every patient.

### Laboratory analysis

Blood samples were obtained after overnight fasting, and before the ingestion of L-thyroxine (L-T4) in those patients who were on L-T4 supplementation. Thyroid stimulating hormone (TSH), free thyroxine (FT4), free triiodothyronine (FT3), antithyroid peroxidase antibody (TPOAb), antithyroglobulin antibody (TgAb), thyrotropin receptor antibody (TRAb), glucose and insulin concentrations were measured in every subject. TSH, FT4, FT3 were measured using electrochemiluminescence technique (normal ranges: TSH 0.27–4.2 mU/l; FT4 11.5–21.0 pmol/l; FT3 3.9–6.7 pmol/l). Estimation of TRAb titers was performed using radioimmunoassay TRAK Human Brahms (normal < 2 IU/l). TPOAb and TgAb were measured by radioimmunoassay (normal ranges: <34 IU/ml and 10–115 IU/ml, respectively). ELISA Assay Kit from Phoenix Pharmaceuticals was used to measure serum NAMPT/visfatin concentration. Glucose level was assessed with the use of Hitachi Cobas e601 chemiluminescent analyzer (Roche Diagnostics) and insulin concentration was assessed using ELISA kit from Phoenix Pharmaceuticals. The estimate of insulin resistance was calculated using homeostasis model assessment (HOMA-IR).

### NAMPT expression studies

In order to obtain biological material for expression studies, the randomly selected peripheral blood samples treated with EDTA were taken from 27 patients with GD, 30 patients with GO and 29 healthy controls. Blood samples were processed immediately after sampling to isolate peripheral blood mononuclear cells (PBMCs), using Pancoll Human reagent (Pan Biotech GmbH.) containing Ficoll 400, as follows: blood was diluted with the same volume of a physiological buffered saline solution (PBS) beforehand, then the Pancoll solution was carefully added without mixing the phases. The samples were centrifuged at 400×*g* for 40 min at room temperature. PBMCs were then retrieved from the boundary layer between Pancoll and the sample layer. This fraction was then washed in PBS twice in order to purify the leukocytes by removing the platelets. RNA was extracted from the leukocytes with a TRIzol^®^ Reagent (Invitrogen, Carlsbad, CA, USA) [26]. Reverse transcription was conducted to obtain complementary DNA (cDNA) using a SuperScript^®^ II Reverse Transcriptase kit with the random primers (Life Technologies). The reverse transcription experimental procedures were carried out according to the manufacturer’s protocol. Quantitative real-time polymerase chain reaction (qPCR) was performed with the StepOnePlus™ Real-Time PCR System (Life Technologies) to quantify human *NAMPT* mRNA expression using Taqman assays (FAM/MGB probes). As the endogenous control, the GADPH gene was used, that was reported to show constant expression in various tissues. Assays were purchased from Life Technologies: NAMPT assay ID Hs00237184_m1, GADPH assay ID Hs03929097_g1. The reaction volume was 20 µl and comprises 10 µl TaqMan^®^ Universal Master Mix II with UNG (2× conc.), 1 µl of assay (20× conc.), 2 µl of cDNA sample and 7 µl of water. The qPCR thermal cycle program includes: incubation at 50 °C for 2 min, an enzyme activation step at 95 °C for 10 min, 40 cycles of denaturation at 92 °C for 15 s and annealing at 60 °C for 1 min. To ensure a high accuracy of the results, all the samples were tested in triplicate. The NAMPT expression was normalized to GADPH and a relative quantitation study was applied (ΔΔ*C*_t_ method). A validation test to determine the PCR efficiency of the target and active reference (endogenous control) has been performed as well. The results showed no discrepancies in amplification efficiencies.

### Statistical analysis

Statistical analysis was performed with MedCalc version 12.1.3.0 (MedCalc Software, Mariakerke, Belgium). Normality was analyzed by D’Agostino-Pearson test. Variables with normal distribution were compared using one-way analysis of variance. If data did not follow normal distribution, comparisons of the analyzed parameters between three groups were performed with the Kruskal–Wallis test. Simple regression analysis was used to find the relationships between them. Furthermore, stepwise multiple regression analysis was employed to investigate the influence of various parameters on *NAMPT* leukocyte expression [age, BMI, FT3, Graves’ orbitopathy (yes/no) or TRAb or TPOAb, HOMA-IR] and NAMPT/visfatin serum concentration [age, BMI, FT3, Graves’ orbitopathy (yes/no), HOMA-IR]. Variables were entered into the model if their associated *p*-values were less than 0.05 and than sequentially removed if their associated *p*-values became greater than 0.2.

All tests were performed two-tailed and were considered as significant at *p* < 0.05.

## Results

**Fig. 1:**
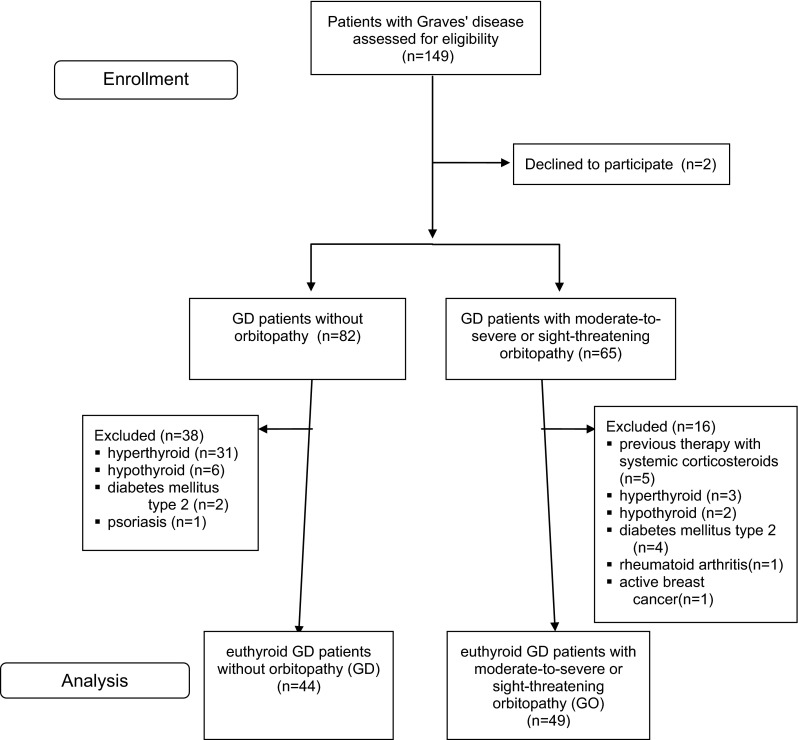
Study flow chart

Of the 149 patients with Graves’ disease, 93 (44 without GO and 49 with GO) were enrolled in further analysis (Fig. 1 presents study flow chart). Three patients had sight-threatening GO (2 had dysthyroid optic neuropathy and 1 had corneal breakdown) and 46 had moderate-to-severe GO. Among GO patients, 35 patients were on levothyroxine therapy after radioiodine ablation (21 patients) or thyroidectomy (14 patients). Fourteen patients achieved euthyroidism during therapy with thiamazole. Twenty-one subjects with Graves’ disease without GO were biochemically controlled by therapy with thiamazole, and 23 subjects were on levothyroxine after thyroidectomy (6 patients) or radioiodine (17 patients). Six patients had inactive GO (CAS < 3) and 43 had active GO (CAS 3–9 patients; CAS 4–16 patients; CAS 5–10 patients; CAS 6–3 patients; CAS 7–5 patients). The control group consisted of 40 healthy subjects (29 females and 11 males) adjusted for age, sex, and BMI with normal thyroid function and negative thyroid antibodies. Clinical and laboratory data of all groups are compared in Table 1.

**Table 1:** Comparison of clinical and laboratory characteristics between the study and the control groups

	GD patients (*n* = 44)	GO patients (*n* = 49)	Controls (*n* = 40)	*p*
Age [year]	42.5 (38–50.5)	51 (40–57.25)	43.5 (37.5–55.0)	0.073
Sex (F-females; M-males)	F 38	F 37	F 29	0.2605
	M 6	M 12	M 11	
BMI [kg/m^2^]	22.35 (20.85–25.23)	23.05 (21.79–27.46)	23.3 (20.85–26.2)	0.1706
Glucose [mg/dl]	93 (89–97.5)	92 (88–97.3)	90 (84–96.5)	0.124
Insulin [μU/ml]	8.7 (6.33–11.95)	9.61 (7.3–12.5)	8.7 (7.2–10.6)	0.246
HOMA-IR	1.95 (1.40–2.73)	2.2 (1.56–2.97)	1.95 (1.43–2.39)	0.276
TSH [μU/ml]	0.9 (0.6–2.2)*	1.09 (0.42–2.38)*	1.76 (1.38–2.57)	0.0008
FT4 [pmol/l]	17.61 (15.4–19.52)	16.36 (13.87–20.10)	15.91 (14.82–17.37)	0.255
FT3 [pmol/l]	5.06 (4.37–5.65)*	4.24 (3.98–5.1)	4.96 (4.85–5.5)*	0.0004
TRAb [IU/l]	9.07 (5.00–15.25)*	8.17 (3.65–16.17)*	0.25 (0.1–0.3)	<0.0001
TPOAb [IU/ml]	220 (41.00–454.5)*	126 (22–195)*	9 (6–13.5)	<0.0001
TgAb [IU/ml]	249.5 (101–508.5)	63 (21.75–208)	10 (10–14)	<0.0001
NAMPT/visfatin [ng/ml]	11.13 (9.55–12.84)*	10.96 (9.56–12.15)*	9.63 (8.75–11.58)	0.0275

Data are presented as medians followed by interquartile ranges given in brackets

* Values followed by the same mark did not differ significantly

**Fig. 2:**
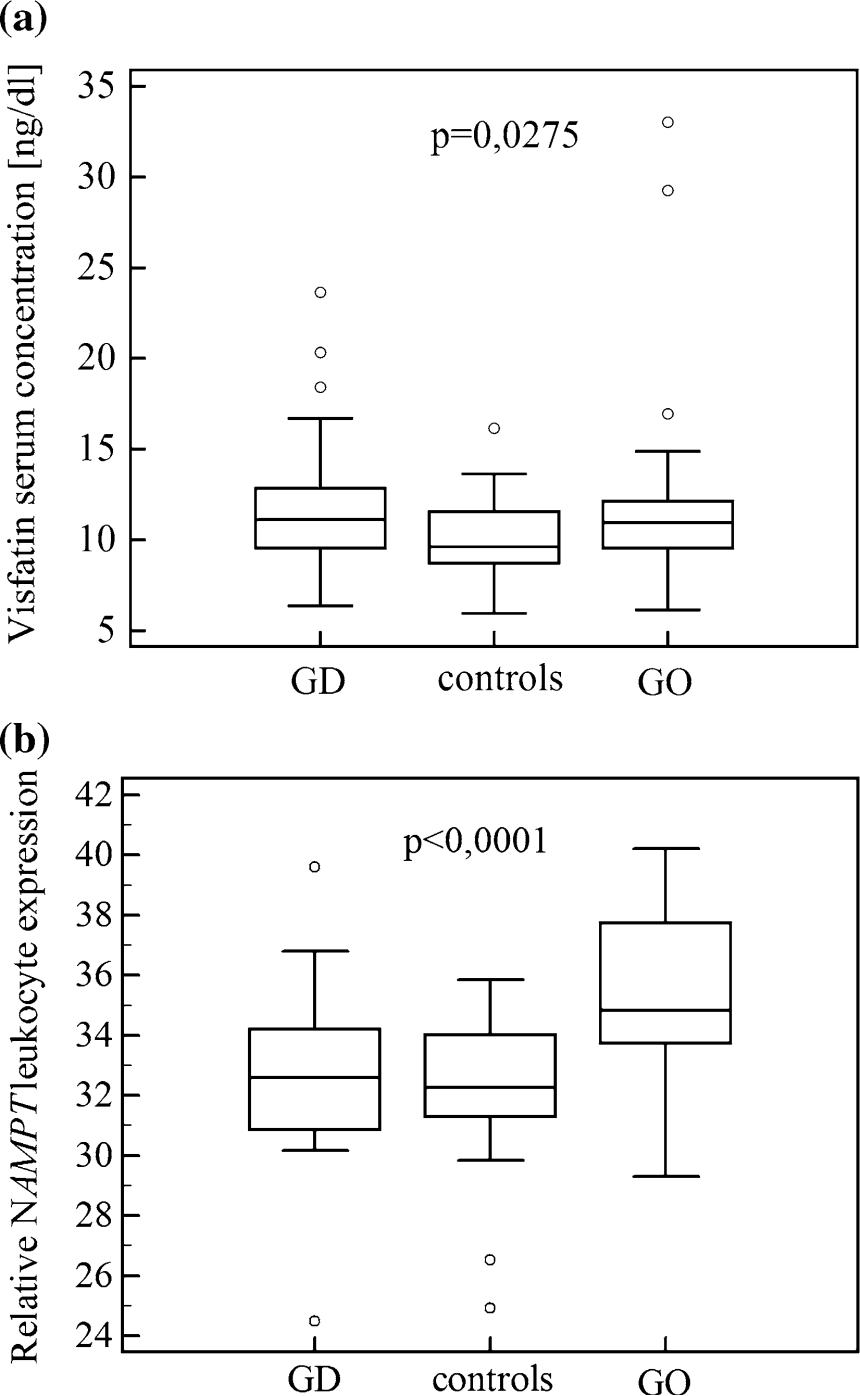
Comparison of **a** visfatin serum concentration and **b** *NAMPT* leukocyte expression between patients with Graves’ disease without (GD) and with Graves’ orbitopathy (GO), and controls. The central box represents the values from the lower to upper quartile (25th to 75th percentile). The middle line represents the median. The *thin vertical lines* extending to the *horizontal lines* (so-called whiskers) extend to a multiple of 1.5× the distance of the upper and lower quartile, respectively. Outliers are any values beyond the whiskers

NAMPT/visfatin serum concentration was higher in GD and GO patients than in the controls (*p* = 0.0275) (Fig. 2). *NAMPT* leukocyte expression was higher in patients with GO (*n* = 30) than in GD patients (*n* = 27) and the control group (*n* = 29) (*p* < 0.0001) (Fig. 2). Study groups and controls did not differ considerably in age, sex, BMI, fasting glucose and insulin levels, HOMA-IR, FT4. Patients with GD and GO had higher levels of anti-thyroid antibodies: TRAb, TPOAb, TgAb. TSH levels were lower in GD and GO patients, and FT3 levels were lower in GO patients.

**Fig. 3:**
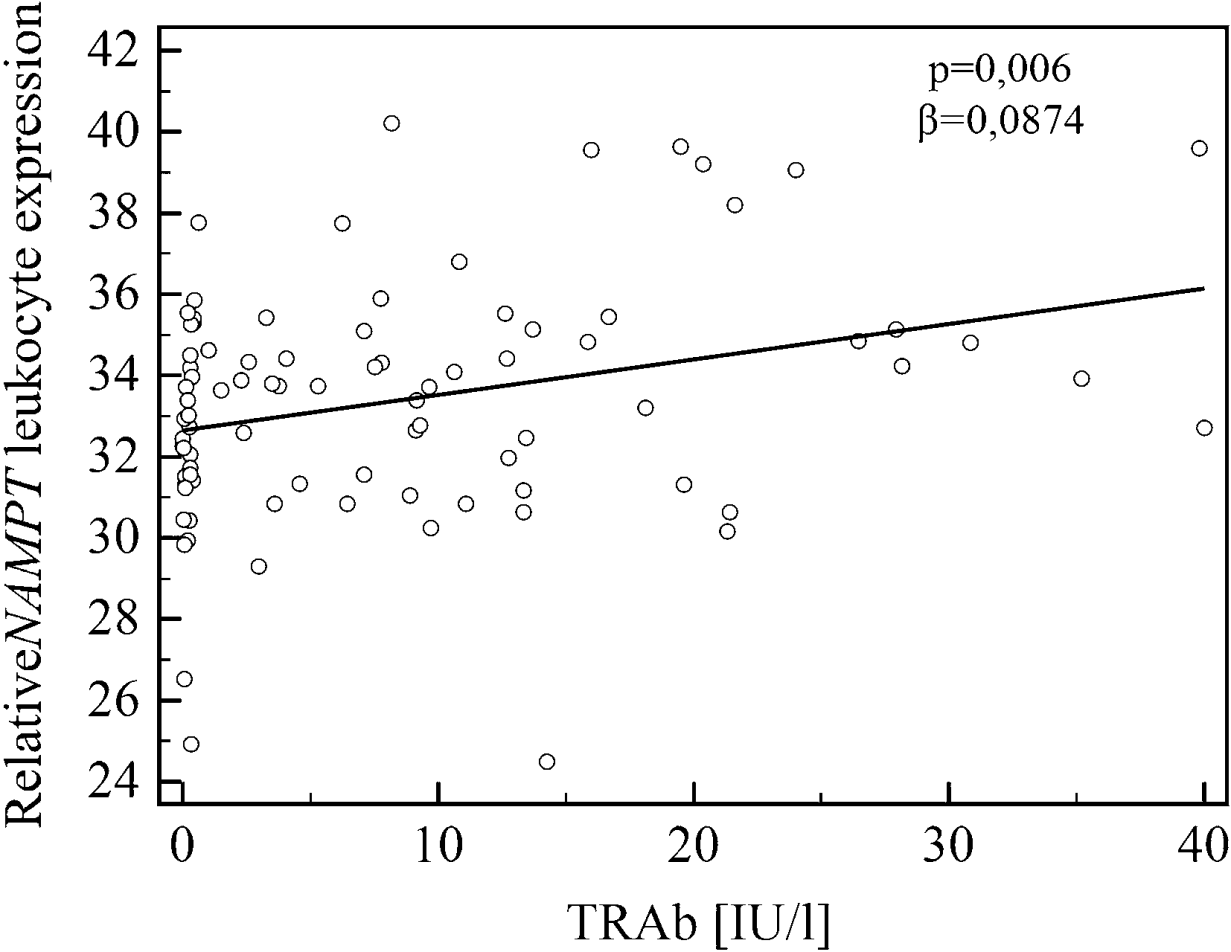
Relationship between *NAMPT *leukocyte expression and TRAb

Simple linear regression analysis revealed that NAMPT/visfatin serum concentration was significantly associated with Graves’ disease (*β* = 1.5723; *p* = 0.021) (Table 2). There was no association between NAMPT/visfatin serum level and age, sex, BMI, TSH, FT4, FT3, TRAb, TPOAb, TgAb, fasting insulin and glucose levels, HOMA-IR, Graves’ orbitopathy, and *NAMPT* leukocyte expression. When *NAMPT* leukocyte expression was used as a dependent variable, simple regression analysis found associations with TRAb (Fig. 3), fasting insulin level, HOMA-IR, Graves’ disease, and Graves’ orbitopathy (Table 2).

**Table 2:** Simple linear regression analysis using visfatin serum concentration or *NAMPT* leukocyte expression as dependent variables

Variable	NAMPT/visfatin serum concentration		*NAMPT* leukocyte expression	
	*β*	*p*	*β*	*p*
Age	0.02139	0.428	0.007012	0.795
Sex	−0.5117	0.506	−0.5311	0.491
BMI	−0.09127	0.275	0.0528	0.55
TSH	−0.2097	0.459	−0.2322	0.433
FT4	−0.01162	0.921	−0.0107	0.926
FT3	−0.4150	0.344	−0.2275	0.589
TRAb	0.03801	0.240	0.08742	0.006
TPOAb	0.001	0.531	0.0005	0.754
TgAb	0.0001144	0.861	0.0003	0.628
Fasting glucose	−0.02293	0.621	0.03793	0.415
Fasting insulin	−0.03293	0.700	0.1816	0.027
HOMA-IR	−0.1373	0.694	0.6993	0.037
Graves disease (yes/no)	1.5723	0.021	1.7235	0.009
Graves orbitopathy (yes/no)	0.7211	0.269	2.4619	<0.001
NAMPT/visfatin serum concentration	–	–	−0.02819	0.742
*NAMPT* leukocyte expression	0.04177	0.742	–	–

In the stepwise multiple regression analysis, we confirmed the association between higher serum NAMPT/visfatin level and Graves’ disease (coefficient = 1.5723; *p* = 0.0212), whereas age, BMI, FT3, HOMA-IR, and Graves’ orbitopathy (yes/no) did not contribute significantly. Also, the stepwise multiple regression analysis revealed GO as an independent predictor of *NAMPT* leukocyte expression after adjustment for the same potential confounders (age, BMI, Graves’ orbitopathy (yes/no), Graves’ disease (yes/no), FT3, HOMA-IR) (coefficient = 2.4619; *p* = 0.0001). In separate stepwise multiple regression analysis, we confirmed the association of *NAMPT* leukocyte expression with TRAb (coefficient = 0.08742; *p* = 0.006), whereas age, BMI, FT3, HOMA-IR did not enter the model.

## Discussion

In the present study, we demonstrated *NAMPT* overexpression in leukocytes of GO patients. In addition, we supported this finding by concordant increased NAMPT/visfatin levels in serum of GD and GO patients. We also found that increased *NAMPT* leukocyte expression is associated with TRAb levels. Furthermore, we confirmed those results in adjusted models. We did not observe the association between GO and serum visfatin level. Since activated leukocytes are not the only source of circulating visfatin, its serum concentration might be influenced by many factors. Therefore, we assumed that NAMPT leukocyte upregulation better reflects its potential immunological properties in GO patients. To the best of our knowledge, this is the first study to describe the serum level of NAMPT/visfatin and its corresponding leukocyte expression in patients with GD and GO. Our results are in agreement to other studies showing increased NAMPT/visfatin level in autoimmune diseases.

The relatively high percentage of moderate-to-severe GO among screened patients with Graves’ disease might be explained by the fact that our Department is the reference center and provides care to patients with moderate-to-severe or sight-threatening GO from Greater Poland.

Pathogenesis of GO is still not fully understood leading to limited therapeutic options [27, 28]. The principal pathogenic hypothesis claims that at least one antigen shared by thyroid and orbital tissue is recognized by autoreactive T-lymphocytes. The recognition is possible due to the strong affinity to antigen-presenting cells. This interaction triggers the cascade of local inflammatory response related to cytokines, chemokines, and growth factors release. Those signals stimulate fibroblasts to secrete glycosaminoglycans and proliferate, as well as prompts differentiation of pre-adipocytes. Glycosaminoglycans promote water intake and together with the increased content of fibroadipose tissue are responsible for clinical features of GO [29]. However, this concept requires further research, in order to recognize cytokines responsible for release of autocrine and paracrine stimuli. Unfortunately, we were not able to analyze *NAMPT* expression in retrobulbar adipocytes in patients who underwent orbital decompression, and that should be considered in future studies. Since the access to the retrobulbar space is invasive, measurement of serum cytokines gives the opportunity to explore new insights, relevant to the pathogenesis of GO.

NAMPT/visfatin/PBEF promotes the development of both T- and B-lymphocytes and stimulates leukocytes for production of pro-inflammatory cytokines such as IL-6, TNF-α, and IL-1β. Its pro-inflammatory effect is also associated with the activation of T cells by up-regulation of co-stimulatory molecules (CD40, CD54, CD80) on monocytes [30, 31]. Th1- and Th2-derived cytokines were found to be elevated in GO patients, but cell-mediated immunity (Th1 cells and associated cytokines, i.e., Il-2, TNF-α, INF) dominates in the early stage of the disease [31, 32]. Notably, IL-6 and TNF-α were predictive factors for the response to orbital radiotherapy. The wide functional profile of NAMPT/visfatin in immunological response would support its potential role as a biomarker or even mediator in pathogenesis of GO. The protein might be involved in activation of signaling pathways responsible for tissue remodeling, and as an adipocytokine might result from enhancement of adipogenesis that occurs in the orbit. The over-production of adipose tissue is one of the histologic hallmarks of GO. Kumar et al. have proved that stimulatory TRAb and TSH are pro-adipogenic factors and cause the increase of adiponectin and leptin genes in orbital pre-adipocytes of GO patients [33]. In another study, they showed that TRAb also stimulates the expression and secretion of Il-6 in GO [34]. Independent association of *NAMPT* leukocyte expression with TRAb levels, which are considered activators of orbital tissue changes, also supports our hypothesis. On the other hand, we cannot rule out the possibility that increased visfatin level reflects the overall activation of the immune system in GO. Another explanation of our findings came with the results of the observational study reporting increased prevalence of DM type 2 and overweight among GO patients [24]. We excluded diabetic patients from the cohort study to avoid the influence of this condition on NAMPT/visfatin concentration, though NAMPT/visfatin is suggested to be a predictive risk factor for DM [35]. Therefore, higher leukocyte expression of NAMPT in GO patients might be related to the observed higher frequency of DM linking metabolic and pro-inflammatory properties of this molecule.

Inhibition of NAMPT/visfatin-related signaling pathways represents possible therapeutic targets. Several NAMPT inhibitors have been synthesized already, and some of them were tested in advanced clinical trials as possible cancer therapeutics [36]. We could not test whether NAMPT was a predictor of GO severity and activity based on our data, but it definitely should be addressed in future studies.

Since FT3 levels were significantly lower in GO patients, we performed the stepwise multiple regression analysis and we did not confirm the influence of FT3 on our findings. Euthyroid GO patients had significantly lower FT3 and TSH, than the healthy control group. An euthyroid state of GO patient was restored pharmacologically (LT-4 replacement therapy, antithyroid medication) as some patients underwent previous radioiodine ablation or thyroidectomy. Our observations are in accordance with the results of the large prospective study recently published by Hoermann et al., who proved that LT4-treated patients had lower FT3, despite lower TSH than non-treated euthyroid patients with autoimmune thyroiditis [37]. Similarly, other authors postulated that LT-4 therapy profoundly alters the physiological TSH-FT3 equilibrium [37–40].

We also observed positive association of *NAMPT* leukocyte expression with fasting insulin level and HOMA-IR confirming previously suggested metabolic role of NAMPT/visfatin [3, 41]. We did not find any relationship between NAMPT/visfatin serum concentration and metabolic parameters. The latter was in accordance with other studies [42, 43]. However, it should be emphasized here that clinical observations have provided contradictory data on the association of NAMPT/visfatin with glucose metabolism parameters [44–46].

## Limitations of the study

The main limitation of the study is its cross-sectional design that limits our ability to reach definitive conclusions about the clinical relevance of increased *NAMPT* leukocyte expression in GO. However, we conducted consecutive enrollment of patients and used strict exclusion criteria, eliminating all known factors potentially influencing NAMPT/visfatin level, such as diabetes mellitus, hypo- or hyperthyroidism, other autoimmune diseases, neoplastic disease. In addition, we confirmed our results in adjusted models.

## Conclusions

In conclusion, increased *NAMPT* leukocyte expression in patients with GO might suggest a presently undefined role in the pathogenesis of GO. Further studies are needed to comprehensively elucidate molecular mechanism behind the observed association.
